# Options for Controlling Type 2 Diabetes during Ramadan

**DOI:** 10.3389/fendo.2016.00032

**Published:** 2016-04-18

**Authors:** Mussa H. Almalki, Fahad Alshahrani

**Affiliations:** ^1^Obesity, Endocrine, and Metabolism Center, King Fahad Medical City, Riyadh, Saudi Arabia; ^2^King Fahad Medical City, College of Medicine, King Saud bin Abdulaziz University for Health Science, Riyadh, Saudi Arabia; ^3^King Abdulaziz Medical City, College of Medicine, King Saud bin Abdulaziz University for Health Science, Riyadh, Saudi Arabia

**Keywords:** hyperglycemia, hypoglycemia, Ramadan, type 2 diabetes, pregnancy, insulin, oral hypoglycemic agent

## Abstract

The Muslim population is about 1.5 billion worldwide. Based on a global diabetes prevalence of 4.6%, it is estimated that there are about 50 million Muslims with diabetes around the world who observe fasting during the month of Ramadan each year. Ramadan, one of the five pillars of Islam, and which takes place during the ninth month of the Islamic calendar, involves fasting from sunrise to sunset. During the fast, Muslims are required to refrain from eating food, drinking, using medications, and smoking from dawn until after sunset, with no restrictions on food or fluid intake between sunset and dawn. Islam exempts people from the duty of fasting if they are sick, or if fasting may affect their health, as fasting for patients with diabetes carries a risk of an assortment of complications, including hypoglycemia, postprandial hyperglycemia, and metabolic complications, associated with dehydration. Nevertheless, a large number of people with diabetes who still choose to fast during Ramadan despite the advice of their doctor, and the permission received from religious authorities thus create medical challenges for themselves and their health-care providers. It is thus important for patients with diabetes who wish to fast during Ramadan to make the necessary preparations to engage in fasting as safely as possible. This review presents a guide to the care of diabetic patients during Ramadan to help them fast safely if they wish to do so.

## Introduction

The Muslim population is about 1.5 billion people worldwide. The approximate number of Muslims with diabetes is around 4.6%; we can estimate that more than 50 million people with diabetes mellitus observe fasting during the month of Ramadan (1, 2).

Healthy adult Muslims practice fasting from dawn until dusk during the month of Ramadan, which varies between 29 and 30 days. The duration of the daily fast may range from a few hours to more than 20 h, depending on the geographical location and season of the year.

During the fast, Muslims are required to abstain from consuming food orally, drinking, using medications, and smoking from dawn until after sunset. People usually eat two meals per day during the month of Ramadan – one after sunset and the other before dawn.

Islam exempts people from the duty of fasting if they are sick or if fasting may affect their health (3). Nevertheless, many people with diabetes still choose to fast during Ramadan against the advice of their doctors, as they perceive themselves as healthy and able to fast.

Numerous studies that evaluated diabetic patients during Ramadan showed that there were little changes in patients’ glycemic control (4, 5). In one study, 76% of patients experienced significant improvements in their fasting blood glucose levels during the fasting period (6). However, the results from the Epidemiology of Diabetes and Ramadan (EPIDIAR) study, which examined 12,243 patients, indicated that 78.7% of type 2 diabetes patients who fasted for at least 15 days during Ramadan were 7.5 times more likely to exhibit an increase in their hypoglycemia, leading to hospitalization during the month of Ramadan when compared to the preceding months (7). Further evidence from a questionnaire survey conducted in Pakistan (which involved 453 diabetic patients) shows that 72.2% of the study population fasted during the month of Ramadan for an average of 25 days (8). Recently, The Multi-Country Retrospective Observational Study of the Management and Outcomes of Patients with Diabetes during Ramadan (the CREED study) of 3,250 patients reported that 94% of patients with type 2 diabetes mellitus (T2DM) fast during Ramadan for a total of at least 15 days, with a mean number of 27 fasting days (9). On the other hand, researchers from the Benghazi Diabetes and Endocrine Centre (BDEC) study indicated that hyperglycemic episodes were reported among 10.7% of 493 T2DM patients who fast during Ramadan (10). Similarly, in a study of 682 patients with diabetes, the hyperglycemic symptoms were felt by 15.41% patients (of a total of 655) with T2DM (11). Also, the EPIDIAR study showed a fivefold increase in the occurrence of severe hyperglycemia during Ramadan in patients with T2DM (7).

Therefore, in people with type 2 diabetes, fasting increases the risk of both hypoglycemia and postprandial hyperglycemia (1, 12). Furthermore, a patient’s decision to cease taking medications, to skip or reduce the doses, or to take the medications at closer intervals without medical supervision increases the risk of such complications. Therefore, this emphasizes the need for close blood glucose monitoring, dietary modifications, and adequate nutrition.

Few studies have compared the efficacy and safety of different antidiabetic agents in fasting patients during Ramadan. In one study, the risk of hypoglycemic events was significantly lower with a dipeptidyl peptidase (DPP)-4 inhibitor than with sulfonylurea (13). In another study, hypoglycemic control was achieved with repaglinide when compared with glibenclamide (14). There is no consensus as to what type of treatment can be given to T2DM patients who decide to fast during Ramadan due to their religious beliefs. Determining the best treatment strategy for T2DM patient who fast during Ramadan is a critical question.

The purposes of this review are to highlight the potential risks that may be associated with fasting during Ramadan, to increase awareness among medical professionals, and to improve their knowledge on how to manage their patients with diabetes who decide to fast during Ramadan.

## Materials and Methods

This review included relevant English-language articles that were identified through searches of major databases, including PubMed, Medline, and Embase (all, 1990–2015), using the keywords “Hyperglycemia, Ramadan, type 2 diabetes, pregnancy, insulin, and oral hypoglycemic agent.” Original research and review articles related to adult patients with diabetes mellitus were considered for the review.

## Effects of Fasting on Glucose Metabolism

In healthy people, insulin secretion decreases in response to the reduction of circulating glucose levels. At the same time, counter-regulatory hormones (glucagon and catecholamines) rise, leading to a breakdown of glycogen (15). Since fasting is prolonged for several hours, the glycogen store becomes depleted, fatty acids release from the adipocytes, and ketones are subsequently generated, which serve as the fuel and sole source of energy for erythrocytes and for vital organs such as the brain. As the fasting hours in Ramadan only extends from dawn till dusk, there is good opportunity to replenish energy stores at sunset and dawn meals. This provides chance for transition from using glucose to fat as the sole source of energy and prevents protein breakdown in muscle. After a few days of fasting, better balance between levels of circulating insulin and the counter-regulatory hormones is achieved; these factors maintain glucose levels within a normal range (16).

In individuals with diabetes, the balance between circulating levels of insulin and counter-regulatory hormones is defective. Therefore, prolonged fasting can lead to hyperglycemia and ketoacidosis as a result of excessive glycogen breakdown and increased gluconeogenesis and ketogenesis; however, ketoacidosis is uncommon in type 2 diabetic patients.

Several studies indicate that fasting during Ramadan neither does worsen a patient’s glycemic parameters nor does it alter the frequency of glycemic complications in patients with type 2 diabetes (17–19). In one study, significant improvements in fasting blood sugar were observed in 76% of patients during fasting (7), whereas other studies did not show this degree of improvement in glycemic control (5, 6). Similarly, the EPIDIAR study showed that hypoglycemia increased about ~7.5-fold in patients with type 2 diabetes, and these patients had at least one episode of severe hyperglycemia requiring hospitalization, 4% of which occurred during Ramadan (7).

## Effects of Fasting on Lipid Metabolism

Several studies have shown no change or little decrease in cholesterol and triglyceride concentrations in patients with diabetes while fasting during Ramadan; conversely, high-density lipoprotein (HDL) cholesterol concentrations tended to increase (18, 19).

## Acute Complications Associated with Ramadan Fasting in Diabetes

### Hypoglycemia

In people with diabetes, Ramadan fasting is a well-known risk factor for hypoglycemia given one’s abstinence from food. Medications and participation in physical activity may increase the propensity for developing hypoglycemia. In a prospective cohort study, the relative risk of hypoglycemia in diabetics who fast during Ramadan was 1.6 when compared with non-fasting periods (20). Similarly, the EPIDIAR study noted that the risk of severe hypoglycemia increased by 7.5-fold in type 2 diabetes patients during Ramadan fasting (7). Finally, in the CREED study, the incidence of hypoglycemia during the month of Ramadan was reported in as few as 8.8% of patients, who reported at least one episode (9).

### Hyperglycemia

In people with type 2 diabetes, prolonged fasting can lead to hyperglycemia and ketoacidosis due to excessive glycogen breakdown and increased gluconeogenesis and ketogenesis. However, ketoacidosis is uncommon in type 2 diabetic patients, while hyperglycemia severity usually depends on the extent to which insulin resistance or deficiency is present. The EPIDIAR study showed a fivefold increase in the incidence of severe hyperglycemia (requiring hospitalization) during Ramadan in type 2 diabetic patients (7). Hyperglycemia may also be attributed to an excessive reduction of medication dosages to prevent hypoglycemia.

### Dehydration and Thrombosis

Dehydration may be due to a number of causes. First, the reduced intake of fluids during fasting may cause dehydration, especially in cases of prolonged fasting and in hot climates. Second, excessive urination due to hyperglycemia can result in volume and electrolyte depletion in the body. Patients may develop symptoms of low blood pressure, especially the one with autonomic neuropathy. A review of the available medical literature indicates that one’s body water status and electrolytes show slight changes during Ramadan. In normal individuals, urine volumes, as well as sodium and potassium concentrations, were lower and urinary osmolality was higher. However, patients’ urinary osmolality concentration was very high in the afternoon, indicating effective water conservation (21).

Studies have shown variable results regarding volume status and electrolyte changes in patients with diabetes during Ramadan. In one study, no significant difference in patients’ total body water was observed before or after the month of Ramadan (22).

Other studies have examined the effects of Ramadan fasting on the hydration status of T2DM patients; a significant increase in urinary osmolality after 1 month of fasting was reported (23). With respect to serum sodium and potassium, no considerable changes occur during the fasting period in Ramadan (24). However, in another study, serum potassium concentration was reduced mainly in the morning, and its amount increased in the afternoon (25). Patient with diabetes have impaired fibrinolytic capacity as a result of elevated plasminogen activator inhibitor type 1 (26, 27). In addition to potentiating platelet function, these patients experience *increases* of tissue expression factors and plasma coagulation factors, such as factor VII, and a decrease in endogenous anticoagulants, such as antithrombin III (28, 29). Thus, diabetes is a hypercoagulable state, and it increases one’s tendency toward thrombosis, while it may also exacerbate the progression of atherosclerosis (30). Contraction of the intravascular space and dehydration during fasting may increase the risk of sinus venous thrombosis, stroke, and retinal vein occlusion (31, 32).

## Pre-Ramadan Medical Assessment and Education

It is worth re-emphasizing the measures that ensure patient safety during fasting in Ramadan. All patients with diabetes who wish to fast for Ramadan should receive a proper medical assessment, educational counseling, and appropriate blood testing 1–2 months before Ramadan to engage in the fast as safely as possible. Advice should focus on the potential risks of fasting, as well as on meal planning and medication regimen modification. The treatment plan should be individualized and educational counseling should include the patient’s family (33).

It is essential to understand that the use of insulin injections during Ramadan is permitted, as it offers no food value (34). Patients should monitor their blood glucose levels several times daily; this is particularly true among those who require insulin. Patients should be educated about the fact that puncturing the skin with glucose monitoring devices is allowed, and it does not affect one’s ability to fast. Furthermore, patients must take fluid liberally during non-fasting hours, and the last meal should be consumed as late as possible; the meal should contain complex carbohydrates, as this will delay digestion and absorption (35). All patients should maintain normal levels of activity and avoid insufficient sleep. Furthermore, they have to understand that they must end their fasting immediately if acute complications, such as hypoglycemia or hyperglycemia, occur (35).

## Management of Patients with Type 2 Diabetes

### Diet-Controlled Patients

Individuals whose type 2 diabetes is well controlled with diet and physical activity should be able to fast without problems, as the risks associated with fasting are low. However, if people eat excessively following the predawn and sunset meals, there remains a potential risk for the occurrence of post-meal hyperglycemia. To prevent this problem, patients should divide their meals into two to three smaller meals during the non-fasting period, rather than consuming one big meal (36). Finally, a person involved in a regular exercise program may modify its intensity and timing to avoid hypoglycemia.

## Patients Treated with Oral Hypoglycemic Agents

### Biguanides (Metformin)

People who take metformin alone should be able to fast safely given that the possibility of hypoglycemia is minimal. However, patients should modify its dose and administration timing to provide two-thirds of the total daily dose, which should be taken immediately with the sunset meal, while the other third is taken before the predawn meal (1, 12, 37). Patients treated with ­slow-release formulations of metformin can take it once daily with the sunset meal (38).

### Thiazolidinediones

Patients should be able to take thiazolidinediones (pioglitazone and rosiglitazone) as usual without dose adjustment, as the risk of hypoglycemia during fasting is low (36, 37). However, combination treatment requires a dose adjustment; one and half of the tablet should be taken immediately at the sunset meal, and the other half must be taken with the meal at dawn (37). These agents are characterized by their slow onset of action, perhaps taking as many as 8–12 weeks. Therefore, these agents cannot be substituted for other oral antidiabetics during Ramadan, as it takes many weeks for their actions to take effect (39).

### Sulfonylureas

Individual treated with this group of drugs should be careful during fasting because they face an increased risk of hypoglycemia. The use of glyburide or glibenclamide may be associated with a higher risk of hypoglycemia than other newer sulfonylureas, specifically gliclazide, glimepiride, and glipizide (40, 41).

Short-acting insulin secretagogues, such as repaglinide and nateglinide, have been shown to be effective with a low risk of hypoglycemia during the fasting period, particularly given their short duration of action. They can be taken twice daily before the sunset and predawn meals. In a study examining type 2 diabetes patients who fasted, it was found that the use of repaglinide was associated with a lower number of hypoglycemia events compared with glibenclamide (14). In another study, 52 people with type 2 diabetes who fasted during Ramadan were randomized to receive either diet alone or a sulfonylurea (glimepiride or gliclazide MR once daily), or repaglinide. The authors concluded that repaglinide was associated with fewer hypoglycemia events when compared with sulfonylureas (42).

As a practical issue, individuals with diabetes who are treated with a once-daily sulfonylurea (such as glimepiride or gliclazide MR) should take the total daily dose with the sunset meal. If an individual takes a shorter acting sulfonylurea (such as gliclazide twice daily), the same daily dose remains unchanged, and the individual should take one dose at the sunset meal and the other at the predawn meal.

In the event that hypoglycemia occurs and persists during the day of Ramadan, the subject should reduce the predawn dose or stop it completely. Long-acting sulfonylureas, such as glibenclamide, should be avoided during fasting.

### Glucosidase Inhibitors

Individuals treated with glucosidase inhibitors (acarbose and miglitol) may safely fast because the risk of hypoglycemia is small. Changes in dose are usually not required (43). These agents decrease postprandial hyperglycemia but have limited efficacy. They are usually used in conjunction with other agents and cause frequent gastrointestinal side effects.

## Incretin-Based Therapy

### DPP-4 Inhibitors

DPP-4 inhibitors, such as alogliptin, saxagliptin, sitagliptin, and vildagliptin, are new oral hypoglycemic agents that act in a glucose-dependent manner and are associated with a minimal risk of hypoglycemia, although they can increase the hypoglycemic effects of sulfonylureas. Patients should be able to take this medication during Ramadan with no dosage adjustment.

Two of these agents have been studied during Ramadan in diabetic patients. In a prospective study of 2,789 individuals with type 2 diabetes, they were inadequately controlled on metformin monotherapy. It was found that the incidence of hypoglycemia with vildagliptin was less when compared to glimepride (44).

Similarly, the VECTOR study, which featured 72 patients, showed no episodes of hypoglycemia with vildagliptin when compared with gliclazide in the setting of combination therapy with metformin in type 2 diabetic patients during Ramadan (45).

In one observational study, vildagliptin as an add-on to metformin was compared with gliclazide in patients with type 2 diabetes during Ramadan fasting. A significantly lower incidence of hypoglycemia was demonstrated with vildagliptin (13). Furthermore, in the VIRTUE study, which was conducted with 1,300 patients with T2DM fasting during Ramadan, vildagliptin demonstrated significantly fewer hypoglycemic events than with sulfonylurea therapy (5.4 versus 19.8%, respectively; *P* < 0.001) (46).

In line with the results from previous studies, the randomized controlled study evaluating vildagliptin compared to gliclazide in patients with type 2 diabetes fasting during Ramadan (STEADFAST) showed that vildagliptin plus metformin is an effective and well-tolerated option in patients with T2DM during Ramadan, as it features a low incidence of hypoglycemia and similar efficacy to gliclazide plus metformin (47).

The use of sitagliptin during Ramadan has been studied in 1,066 patients with type 2 diabetes. It was found that the risk of hypoglycemia was significantly lower with sitagliptin compared to those who remained on a sulfonylurea (48). More recently, a study involving 848 patients who fasted during Ramadan showed that sitagliptin reduced the risk of hypoglycemia by approximately 50% when compared to sulfonylureas (49).

### Glucagon-Like Peptide-1 Receptor Agonists

This class of agents, which includes exenatide and liraglutide, is not independently associated with hypoglycemia; therefore, it may be suitable for Ramadan. However, these agents can increase the risk of hypoglycemia when combined with sulfonylureas and insulin; hence, the sulfonylurea or insulin dose may need to be reduced. It is important to note that when these agents are used alone during Ramadan, they do not require any dose adjustments (50). Glucagon-like peptide-1 (GLP-1) receptor agonists require dose titration, and they are associated with nausea and vomiting, particularly as therapy is initiated; this could be a limiting factor during fasting. Currently, there is not enough evidence regarding the use of these agents during Ramadan, although studies that did not assess patients during Ramadan did show that these agents cause fewer hypoglycemic events (similar to monotherapies) when compared to conventional treatments. Recently, The Treat 4 Ramadan study was the first randomized controlled trial to compare liraglutide with sulfonylureas use in top of metformin among 99 T2DM patients. That study showed that there were significantly fewer hypoglycemic episodes in the liraglutide arm. The author concluded that liraglutide is well tolerated, and it may even be an effective therapy when used in combination with metformin during Ramadan (51).

### Sodium Glucose Co-Transporter 2 Inhibitors

Sodium glucose co-transporter 2 inhibitors represent new agents with a unique mode of action that improves glycemic control by decreasing the renal reabsorption of glucose; these agents do not cause hypoglycemia (52). They offer a safe option for patients who fast during Ramadan without compromising glycemic control. However, there are no studies of these agents during periods of fasting. Trials to examine the safety and tolerability of these agents during Ramadan would be beneficial.

### Insulin

The glycemic control of patients with type 2 diabetes during Ramadan should be individualized. Patients treated with insulin usually face the same problems as those with type 1 diabetes, except for the fact that the incidence of hypoglycemia is lower. Moreover, patients with T2DM also need to maintain a high enough level of basal insulin to prevent fasting hyperglycemia. An effective strategy for patients using multiple daily injections would be to administer long-acting insulin with a meal at sunset and to reduce the dose by 20% to reduce the risk of hypoglycemia (53). For insulin taken before meals, patients should omit the afternoon dose and take the morning dose at the sunset meal; they should also take half of the evening dose at dawn.

Rapid-acting insulin analogs (when compared to regular human insulin) before meals is associated with a lower incidence of hypoglycemia and minimal postprandial hyperglycemia (53–55). The use of these newer insulin analog preparations during fasting may be more useful, as regular human insulin has a long-lasting peak with a high possibility of late postprandial hyperglycemia (55).

Another important note is that when using premixed insulin, the patient should take the morning dose at the sunset meal and half of the evening dose at dawn (50, 53, 56). If patients take the once-daily premixed insulin before Ramadan, then the same dose should be taken before the sunset meal (57), with further insulin dose titration performed based on home blood glucose data.

## Diabetic Medication Adjustment during Ramadan

The summary and recommendations for adjusting therapy during Ramadan in patients with type 2 diabetes are illustrated in Table 1.

**Table 1:** **Recommended changes to the treatment regimen in patients with type 2 diabetes who fast during Ramadan**.

Treatment	Modification needed during Ramadan
Patients on diet and exercise	Distribute meals into 2–3 smaller meals, modify exercise intensity and timing, and ensure adequate fluid intake
Bigunide (metformin)	Immediate-release formulations: two-thirds of the total daily dose should be taken immediately with the sunset meal and the other third taken before the predawn meal
	Slow-release formulations: can be taken once daily with the sunset meal
Thiazolidinediones	No change needed
Sulfonylurea	Once-daily sulfonylurea (such as glimepiride or gliclazide MR): the total daily dose should be taken with the sunset meal
	Shorter-acting sulfonylurea (such as gliclazide twice daily): the same daily dose remains unchanged, and one dose should be taken at the sunset meal and the other at the predawn meal
	Long-acting sulfonylurea (such as glibenclamide): these agents should be avoided
Short-acting insulin secretagogues (repaglinide and nateglinide)	No change needed
Glucosidase inhibitors	No change needed
DPP-4 inhibitors	No change needed
GLP-1 analogs	No change needed
Sodium glucose cotransporter 2 inhibitors (SGLT2 inhibitors)	There are no studies of these agents during periods of fasting, so their use is not recommended, or they should be used with caution
Insulin	Multiple daily injections: long-acting insulin at sunset with 20% reduction of the dose. For pre-meal insulin, omit the afternoon dose and take the morning dose at the time of the sunset meal, and take half of the evening dose at dawn
	Premixed insulin: morning dose should be taken at the sunset meal and half of the evening dose should be taken at dawn

## Management of Hypoglycemia during Fasting

Diabetic patients who insist on fasting during Ramadan should be aware of the risk hypoglycemia; they should receive appropriate advice to decrease this potential risk. The most crucial issue is that the patients should have ability to monitor their blood glucose levels several times daily, which is especially critical for patients who require insulin. Those with diabetes should eat a healthy, balanced diet, and avoid overeating, especially at sunset. It is also advisable that the last meal be taken as late as possible and with an adequate amount of fluid during the non-fasting hours. Moreover, diabetic patients should maintain normal levels of activity, as excessive physical activity – particularly during the few hours before sunset – may lead to hypoglycemia. It is essential that patients end their fasting immediately if hypoglycemia [blood glucose of <60 mg/dL (3.3 mmol/L)] occurs, as their blood glucose levels may drop further if they delay treatment. Obviously, given the increased risk of hypoglycemia during Ramadan, one should adjust the treatment regimen accordingly.

## Lifestyle Interventions and Education during Fasting

Ramadan-focused education and lifestyle modifications should be re-enforced before and during Ramadan to minimize the risk of hypoglycemic events and the occurrence of post-meal hyperglycemia. In one study, patients who did not attend an educational program had a fourfold increased risk of hypoglycemic events, whereas those who attended the program had a significant decrease in these events (33). It is essential that diabetic patients who want to fast should continue to monitor their blood glucose levels several times throughout the day; this is particularly critical for those who require insulin.

It is important that diabetic patients eat a healthy balanced diet and that they choose foods with a low glycemic index (such as complex carbohydrates), which can help to maintain blood glucose levels during fasting. Moreover, it is crucial to consume adequate fluid to prevent dehydration. Physical activity is encouraged, especially during non-fasting periods. Prayers after sunset should be considered as part of the daily exercise program. Family members should receive adequate and appropriate education on self-care and diabetes management during Ramadan to minimize any negative effects that might result from fasting.

## Pregnancy and Fasting during Ramadan

Pregnancy involves a state of increased resistance and insulin secretion. In healthy pregnant women, fasting blood glucose levels are low, while postprandial glucose and insulin remain high when compared to non-pregnant women. Pregnant Muslim women are exempt from fasting during Ramadan; however, some choose to fast due to their personal beliefs or religious commitments.

Fasting during pregnancy is believed to carry a high risk of morbidity and mortality to both the fetus and the mother (58). Therefore, it is strongly recommended that pregnant women not fast during Ramadan. The management of pregnant patients during Ramadan includes providing an appropriate pre-Ramadan assessment and education to ensure that the required diet and insulin dose adjustment are in place. These patients should also be monitored more frequently.

## Diabetes Management after Ramadan

A 3-day festival “Eid ul-Fitr” follows the month of Ramadan. This is usually characterized by the sharing of food and sweet beverages. The management of Muslim people with diabetes during this period of time continues to be a challenge, as many patients overindulge in eating and drinking. Diabetic education during this time is an essential tool to prevent potential complications such as hypoglycemia, hyperglycemia, and diabetic ketoacidosis. It is critical that diabetic patients are well informed to monitor their blood glucose levels several times daily, to avoid overeating, and to adjust their medications; patients may be changed to their previous regimen if their glycemic control was optimal before the month of Ramadan (53).

## Conclusion

Managing diabetic patients during Ramadan continues to be a challenge. It is crucial that health-care providers are able to offer advice and counseling to diabetic patients to help them fast safely, if they wish to do so. It is important to individualize each patient’s management plan depending on his or her diet and lifestyle, medications, risk of hypoglycemia, and glycemic control, and to minimize the complications associated with fasting. The practical management of these patients is made based on the knowledge of carbohydrate metabolism and on hormonal changes during pregnancy, as well as on the pharmacology of various antidiabetic drugs. In general, the risk of hypoglycemia and hyperglycemia in patients with type 2 diabetes is not overly common, and it is associated less severe consequences.

Further studies are needed to help expand our knowledge concerning all the factors that affect blood glucose control during this period.

## Author Contributions

Both authors contributed equally to this work, and they discussed the implications and commented on the manuscript at all stages.

## Conflict of Interest Statement

The authors declare that the research was conducted in the absence of any commercial or financial relationships that could be construed as a potential conflict of interest.
